# Medicine as a Profession

**Published:** 1904-09-03

**Authors:** 


### Medicine as a Profession.

The profession of Medicine offered no very enchanting prospect when the Church, the Bar, the Services, and Medicine were alone open to the sons of middle-class parents. The training began by apprenticeship either with a large premium and little work, as in the case of the hospital apprentices, or with a small premium and a great deal of menial work as was more commonly the case. In those days a boy became a physician, a surgeon, or a general practitioner. The physician must necessarily have obtained a University degree ; he lived a life of learned ease and depended wholly upon auscultation and percussion for his knowledge of disease. The surgeon spent his time "'Mongst horrid shapes and shrieks and sights unholy." The operating theatre was a shambles and the wards were stinking. The general practitioner held a very inferior position in the public estimation and, of course, with important exceptions, was little better than a tradesman. It is no wonder, therefore, if the profession of Medicine failed to attract a very high-class recruit and if the better men drifted into the Church or went to the Bar. Gradually, however, all this has changed. The physician has improved his knowledge and sharpened his wits by a devotion to pathology in all its branches but more especially by studying bacteriology and pathological chemistry. The surgeon has emerged from the thraldom in which he was held by the physicians and has become the true autocrat of the profession. The blessings of anæsthesia and the security afforded by Listerian methods have attracted a wholly new type of men to surgery. It was sufficient formerly if a surgeon were dexterous and callous. To-day he has perfected his dexterity but he is as keen, as alert intellectually, and as highly trained as a successful barrister. University men who would have entered the Church or gone to the Bar with a prospect of obtaining high place are now drawn to surgery. But it is not everyone who is able or fitted to become a physician or a surgeon ; the majority must ever remain as general practitioners. Still, even as general practitioners their lot is greatly mitigated ; in towns there are constantly new openings for a young man whilst the distress and grinding poverty is certainly less than it used to be. In the country if large houses are fewer and the farmers are less lavish than of old, the labourers are in a better position and the population is increasing, if not in numbers at any rate in worldly prosperity. Medical clubs exist and are abused in England, but they are not as rampant as in many of the colonies, whilst the position of the medical practitioner in England is infinitely superior to that of his colleagues in any European country at the present time. New methods of locomotion and of communication have reduced the sense of isolation and consequently the responsibility of the general practitioner and the suppression of many epidemic diseases have lessened the chance of his being worked to death. But there are other openings for a newly-qualified practitioner which were not in existence a few years ago. He may choose to become a specialist ; the Navy, the Army, and the Colonial medical service offer advantages to those who for any reason are unwilling to enter upon the burden of general practice and are not wedded to their native country. Sanitary science, whether under the Local Government Board or the various county councils, yield good opportunities to those who prefer to study disease in the abstract rather than in the individual or who are bitten with the desire *rerum cognoscere causas.* And with all these advantages there is the further one that the parting of the ways does not come until after several years and when the boy has already become a man. Whatever his destiny the first five years of his training are identical, and it is not until he has become legally qualified to practise every branch of his profession that he can pursue the special bent of his inclination. In these five years he has seen many men and many methods and he has discussed them with all the freedom of youth. He has been made to feel responsibility too and he has learnt how slight an error of judgment may lead to the most disastrous consequences. By the end of his training, therefore, he is competent to choose his path in life for he knows wherein his strength lies. Of course, the intending medical student should look well ahead, and it is especially necessary that he should have a disposition which will accord well with his future as a medical man ; but in modern medicine there are so many fields and special branches of work that it will not be common for anyone, provided he is prepared to do his share of hard work, to fail in the quest for an opening which is suited to his natural bent.

